# Creation and Verification of a Machine Learning-based Model for Predicting Breast Cancer Risk in Patients with BI-RADS 4 Breast Lesions

**DOI:** 10.2174/0115734056463694260528161223

**Published:** 2026-06-10

**Authors:** Jiahua Zhao, Bo Liang, Jiaxin Yan, Jianting Zheng, Shurong Chen, Haiqing Chen, Jun Zhou

**Affiliations:** 1 Department of Medical Ultrasound, Affiliated Hospital 2 of Nantong University, Nantong, China

**Keywords:** Breast lesions, Breast imaging reporting and data system category, Elasticity imaging techniques, Machine learning, Shapley additive explanation, Nomogram

## Abstract

**Introduction:**

To develop and validate a machine learning-based model for predicting the risk of breast cancer occurrence in BI-RADS 4 patients.

**Methods:**

216 breast lesions from 212 patients were included for retrospective analysis. The training set (151 cases) and the validation set (65 cases) were randomly divided from the whole data set at a ratio of 7:3. Use logistic as well as LASSO regressions to identify independent risk factors. Subsequently, eight ML models were constructed. After a comprehensive comparison, the optimal model was selected and visualized using the SHAP algorithm.

**Results:**

Through feature selection, six parameters were identified as independent risk factors. The Emax-2 shell cutoff (104.71 kPa) demonstrated the highest diagnostic efficacy for the 4a subgroup. Among the eight ML algorithms, the random forest model exhibited potential overfitting risks(AUC=1.00), whereas the LR model demonstrated superior stability. Consequently, the LR model was selected as the predictive model, and a nomogram was constructed based on it.

**Discussion:**

In this study, the LR model enhanced the capacity to identify BI-RADS 4 lesions as BC. However, the study is a single-center study with a relatively small sample size, which brings certain restrictions to the clinical application of the model.

**Conclusion:**

The LR model is the best choice for predicting BC incidence in BI-RADS 4 lesions, which can help clinicians improve BC identification and treatment at an early stage.

## 1 INTRODUCTION

Breast cancer (BC) is currently the leading malignant tumor affecting women globally, and its prevalence, along with death rates, rises year after year [1]. Better prognosis and higher survival rates for BC patients can be achieved through early identification and prompt intervention.

With a 2%–95% probability of malignancy, Breast Imaging Reporting and Data System (BI-RADS) 4 breast lesions must be closely regulated. According to the guidelines, a needle biopsy should be performed on all of these lesions to confirm their pathological condition [2]. This approach not only increases patients' psychological and financial burdens but also wastes medical resources.

Innovative approaches to BC early screening have been made feasible by advancements in imaging technology. Mammography remains a vital tool for early detection. However, it has a low sensitivity for identifying lesions in thick breast tissue, which is frequent in Asian women [3]. As a result, breast ultrasound is recommended for BC screening by the Chinese Society of Radiation Oncology Imaging [4]. Widely used in clinical settings and well-liked by patients for its convenience, low cost, radiation-free, and non-invasive, breast ultrasound's imaging effect is affected by certain factors, such as noise levels, image contrast, and so on [5].

Recently, emerging ultrasound techniques have significantly advanced, improving the efficiency of BC diagnosis, and ultrasonic elastography (UE) has gained popularity in clinical applications due to its simplicity. Strain elastography (SE) and shear wave elastography (SWE) are two different types of techniques applied in UE. By exerting continuous pressure and using color elastic diagrams that rely on operators’ skill and technique, SE evaluates tissue stiffness [6]. Based on the principle of the ‘Mach cone’, SWE is an innovative quantitative elastography method that measures tissue's Young's modulus hardness value to account for elastic fluctuations; higher values indicate harder tissue. As a reproducible technique, SWE can evaluate tumor progression [7]. However, SWE is less effective for evaluating deep lesions and similarly relies on operators’ skill. Zhou *et al*. [8] observed that increased tissue stiffness often occurred around tumors, manifesting as a “stiff rim” sign on elastography. Research indicates that the “stiff rim” sign correlates with collagen fiber proliferation and increased stiffness at the margins of BC lesions, whereas benign tissues exhibit uniform internal growth with the overall lesion stiffness distributed homogeneously. The “stiff rim” sign can be used to indirectly predict the invasive growth and prognosis of BC at an early stage. Consequently, several researchers use the stiffness distribution features in elasticity images to distinguish between benign and malignant tumors. Previous studies focused solely on the breast lesion itself. In recent years, an increasing number of studies have focused on the hardness of the surrounding breast tissue [9]. Studies revealed that the Emax-2 shell had the most diagnostic value in distinguishing benign from malignant breast lesions based on the hardness value of the lesion and its surroundings [10].

In general, machine learning (ML) refers to computer techniques or models that use empirical data to improve performance or produce precise predictions [11]. ML is widely applied in ultrasound, MRI, and digital breast tomosynthesis; it can give us more objective and accurate predictive methods, from the benign and malignant nature prediction of breast lesions, all the way to the prediction of BC molecular subtypes [12], assisting clinicians in detecting possible trends of disease progression, promoting personalized treatment strategies, and improving treatment outcome. Ma *et al*. [13] constructed five ML models to predict BC molecular subtypes, concluding that the decision tree (DT) model demonstrated the best performance in distinguishing triple-negative BC, providing additional reference value for clinicians identifying BC molecular subtypes. Zuo *et al*. [14] built eleven ML models to predict BC recurrence risk, with the Adaptive Boosting (AdaBoost) model exhibiting the highest predictive performance, aiding physicians in clinical decision-making. LR is often used as a baseline comparison due to its simplicity, efficiency, and interpretability, making it one of the preferred tools for solving classification problems [15]. XGBoost combines several weak learners to create a strong learner. It shows great promise for developing illness risk prediction models, locating possible treatment targets, and carrying out accurate patient stratification. An efficient regularization approach built into Light Gradient Boosting Machine (LightGBM) improves model fitting performance while reducing the danger of overfitting. Usually used for binary classification tasks, SVM is a supervised learning model. It performs remarkably well in situations with unclear data boundaries and is particularly good at handling high-dimensional data. Building upon the optimal model results from previous BC studies and integrating the strengths of other ML algorithms, this study combined clinical characteristics, conventional ultrasound, and elasticity features. It specifically investigated the diagnostic value of 2mm peritumoral tissue elasticity parameters for detecting BC in BI-RADS 4 lesions. LR, DT, RF, GNB, SVM, AdaBoost, XGBoost, and LightGBM algorithms were used in model creation.

The SHAP algorithm refers to a visualization tool for interpreting ML model prediction results. Its core concept is derived from the Shapley value in game theory, which quantifies each participant's contribution to collaboration, thereby ensuring a fair distribution of benefits. The SHAP algorithm adapts this concept to ML by calculating the Shapley value for each feature, quantifying its contribution to the model's prediction. It assists in determining which features are most important to the predictive model by offering both local and global explanations. The SHAP algorithm has a broad applicability and can be applied to multiple ML models, including neural networks, tree models, linear models, *etc*. [16].

Therefore, this study performed a screening of independent predictors for the occurrence of BC in BI-RADS 4 patients based on clinical basic characteristics, conventional ultrasound features, and combined with elastography features. Then, the development and comparisons of four risk predictive models based on ML were conducted for the sake of performance evaluation. The optimal model was selected, and the SHAP algorithm was utilized for visually interpreting the model's predictive results.

Briefly, this study intended to provide an emerging way for early prediction of the risk of BC in BI-RADS 4 patients, offering corresponding guidance and assistance to clinicians for making personalized treatment plans and further optimizing clinical decision-making.

## 2 MATERIALS AND METHODS

### 2.1 Patient Selection

212 female patients were retrospectively selected from among those who met the inclusion criteria and were hospitalized at Nantong First People’s Hospital from October 2023 to June 2025, comprising a total of 216 breast lesion cases (45 lesions were excluded).

Inclusion Criteria: Patients diagnosed with BI-RADS 4 lesions who underwent routine ultrasound and elastography examinations before surgery/biopsy; No prior surgery, chemotherapy, or endocrine therapy; Complete case records; Available histopathology reports.

Exclusion criteria: Pregnant or lactating patients; Patients with severe organ dysfunction; History of other malignant tumors (non-primary lesions); Unacceptable elastography image quality.

**Fig. (1):**
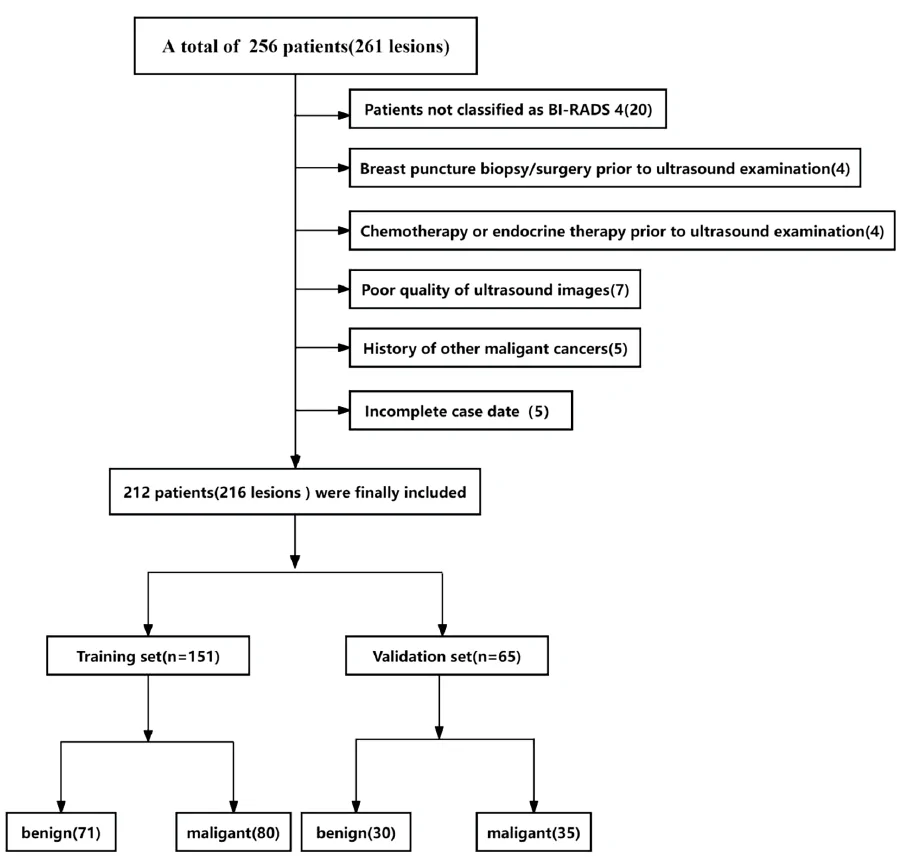
The flowchart of patient inclusion and exclusion.

Using pathological findings as the gold standard, all lesions were categorized into benign and malignant groups. Figure **1** is the flowchart for patient inclusion/exclusion.

### 2.2 Sample Size

Concerning Kendall's requirements for sample size in multivariate analysis, the sample size of this research should be 5-10 times the number of independent variables. There were 22 variables in this study; hence, a sample size of 110–220 cases was needed. The 22 variables comprised: Basic clinical data: age, BMI, menopausal status; Conventional ultrasound parameters: maximum diameter, orientation, echo pattern, shape, margin, hyperechoic halo, posterior acoustic, microcalcification, Alder classification; Elastography parameters: Mean Young's modulus hardness value (Emean), Maximum value (Emax), Minimum value (Emin), Standard Deviation (Esd) and Mean Young’s modulus hardness value of the 2 mm Shell (Emean-2shell), Emax-2 Shell, Emin-2 Shell, Esd-2 Shell, the “Stiff Rim” Sign, and SE Score. The final calculated sample numbers were between 132 to 264 cases, considering a 20% loss rate when the sample was taken with systematic sampling error. The number of samples collected by the research was adequate for the research sample size.

### 2.3 Conventional Ultrasound and Elastography Examination

The Mindray Resona I9 L14-3U linear array probe (frequency 3 - 14 MHz) was used to perform the examination. Place the patient in a lying position, and abduct both upper limbs 90°angle to fully expose the examination area (including both breasts and axillae), facilitating whole-part scanning and ensuring the accurate localization of breast lesions. If the size of the breasts caused tilting, the contralateral lateral decubitus position may be used to incline the body slightly.

Conventional Ultrasound Examination: Make a judgment of malignancy risk based on the orientation of the nodule, shape, margin, echo type, posterior sound characteristics, Alder classification, and whether there is microcalcification. The evaluation adheres to the V5 requirements of the American College of Radiology (ACR) BI-RADS [17].

SE examination: Change to elastography mode once the lesion was clearly seen on conventional ultrasound. Scale the sample frame to about twice the area of the lesion and adjust its location to center the lesion inside the frame. The probe should be positioned perpendicular to the skin. To score the hardness of the lesion, freeze the frame and save the image when it has been stabilized. Elasticity scoring employed the modified 5-point scale proposed by Itoh *et al*.[18]: Score 1: Entire or predominant green display; Score 2: Mosaic pattern of green and red; Score 3: Green periphery with red center; Score 4: Predominantly red or minimal green within red; Score 5: entire lesion and surrounding tissue appeared.

SWE examination: When the breast lesion’s maximum diameter is visible on 2D ultrasound, perform SWE. In elastography images, hard tissue was shown as red, soft tissue as blue, and intermediate tissue as green. Lightly touch the probe to the skin without applying pressure, maintaining a vertical angle with the skin. Click the elasto button and set the region of interest (ROI) so that most of the lesion and normal tissue around it are covered. Let the patient hold their breath. Launch the shear wave software on the Mindray Resona R9 ultrasound system. Utilizing the Motion Stability Index (M-STB index) and Reliability Index (RLB index) for real-time monitoring and quality control of shear wave stability in breast lesions. Ensure image freeze occurred under conditions of low motion interference (M-STB index 4 or 5 stars) and high reliability index (RLB index > 95%). Press the Measure key and select Young's modulus to trace the tumor region. The system automatically calculated Emean, Emax, Emin, and Esd within the lesion. Adjust the knob to select a 2mm shell size. The system would automatically calculate Emean-2 shell, Emax-2 shell, Emin-2 shell, and Esd-2 shell. Perform three measurements for all parameters and take the average.

### 2.4 Image Acquisition

Performed jointly by two physicians who had undergone specialized training and possess over five years of ultrasound experience. Another highly experienced senior physician performed a second review in cases when the diagnostic results were not consistent. After the discussion, a consensual conclusion was achieved.

### 2.5 Predictor Variable Selection and Model Construction

In this study, to ensure reproducibility among features, parameters with an intraclass correlation coefficient (ICC) ≥0.75 were included in the feature selection process. Within the training set, univariate LR, Lasso regression, and multivariate LR were successively applied to the dataset to identify independent risk factors associated with BC occurrence in BI-RADS 4 patients. Lasso analysis retained variables with non-zero regression coefficients under the optimal regularization parameter (lambda. 1se), avoiding multicollinearity among variables. Lasso analysis achieved feature selection through L1 regularization, simplifying the model and enhancing interpretability while effectively controlling overfitting. Ten-fold cross-validation enhanced model stability and reliability through iterative training and validation cycles, aiding in regularization parameter optimization. Subsequently, eight ML models were constructed. Hyperparameter optimization was performed using grid search within ten-fold cross-validation to improve model generalization capability.

### 2.6 Model Evaluation and Interpretability

Evaluate model performance using validation set data. Assess model performance comprehensively through AUC, accuracy, sensitivity, specificity, PPV, NPV, and F1 score. Employ DeLong's test to evaluate diagnostic efficacy.

Through comprehensive comparison, select the optimal ML model and apply the SHAP algorithm to explore its interpretability.

### 2.7 Predictor Variable Selection and Model Construction

Statistical analysis was performed using SPSS version 27.0, R version 4.2.3, and Python version 3.11.4. For normal continuous data, the data were displayed as the mean ± standard deviation (SD), and differences between the two groups were analyzed by the independent-samples t-test. For non-normal continuous data, the data were given as median (M) and interquartile range (Q1, Q3), and the Mann-Whitney U test was employed for group comparisons. Categorical variables were represented as n (%), and the Chi-square test was used to compare groups.

The predictive accuracy of the nomogram model was assessed by the calibration curve, and its calibration was evaluated using the Hosmer-Lemeshow goodness-of-fit test. Decision curve analysis (DCA) was employed to evaluate the clinical utility of the nomogram model, quantifying its net benefit across a range of threshold probabilities. ROC curves were generated using the “pROC” and “ggplot2” packages, calibration curves using the ‘rms’ package, and DCA using the “ggDCA” package. A p-value < 0.05 was considered statistically significant.

### 2.8 General Clinical Data and Pathological Findings

We gathered 216 breast lesions from 212 women. The patients’ ages ranged from 21 to 86 years, with the mean being 52.00(41.00, 61.00) years. Lesion sizes varied from 4.00 to 50.00 mm, and the mean maximum diameter was 16.00 (10.50, 22.00) mm. Out of the overall 216 lesions, 101 (46.8%) were benign and 115 (53.2%) were malignant. The included patients were categorized by BI-RADS as 125 4a, 55 4b, and 36 4c. There were 93 (74%) benign cases and 32 (26%) malignant cases in the 4a subgroup; 6 (11%) benign cases and 49 (89%) malignant cases in the 4b subgroup; and 2 (6%) benign cases and 34 (94%) malignant cases in the 4c subgroup. Pathological findings revealed 101 benign lesions, including 49 fibroadenoma, 22 adenosis, 10 adenosis with fibroadenoma, 9 intraductal papilloma, 4 inflammatory lesions, 2 adenosis with intraductal papilloma, 2 benign tumors, 2 cysts, and 1 hyperplasia lesion. Pathological findings revealed 115 malignant lesions, including 92 cases of invasive ductal carcinoma, 15 cases of ductal carcinoma in situ, 4 cases of invasive lobular carcinoma, and 4 cases of invasive special type cancer.

## 3 RESULTS

### 3.1 Baseline Data

Randomly set 7:3 for the training set (n=151) and validation set (n=65). Unlike the training set, which had 71 benign and 80 malignant lesions, the validation set had 30 benign and 35 malignant ones. The baseline characteristics of the two groups did not differ significantly (*P* > 0.05). The corresponding data distribution was presented in the following Table **1**. The distribution of baseline data for the training set is given in the following Table **2**.

### 3.2 Univariate Logistic Regression Analysis

The training set data used 22 parameters for univariate logistic regression. The results revealed that, except for 4 parameters (BMI, echo pattern, Emin, and Emin-2 shell), the remaining 18 parameters all had *p*-values <0.05, indicating statistically significant differences (Table **3**). For patients with BI-RADS 4 breast lesions, these parameters serve as independent indicators of BC risk.

### 3.3 Predictive Variable Selection

LASSO regression analysis was used to further screen variables based on univariate logistic regression analysis to successfully limit multicollinearity across parameters, given the large number of elastic parameters included. The values for two sets of lambdas, lambda.min (0.018) and lambda.1se (0.047), were obtained from ten-fold cross-validation. To improve the model generalization, lambda. 1se (0.047) was chosen as the best parameter for this study. Preliminary screening of 10 possible predictor variables: age, menopausal status, orientation, shape, margin, Emax-2 shell, hyperechoic halo, microcalcification, “stiff rim” sign, and SE score. The detailed process of variable selection using LASSO regression was represented in Fig. (**2**). Multivariate stepwise logistic regression analysis was done with the ten parameters mentioned above, and at the end, it was shown that 6 variables were independent predictors of BC risk in patients with BI-RADS 4 breast lesions, namely age, orientation, shape, microcalcification, Emax-2 shell, and SE score (Table **4**).

Among the eight SWE parameters included, the Emax-2 shell was ultimately retained for further investigation of its diagnostic efficacy for BI-RADS 4a, 4b, and 4c subgroups in this study. This study concluded that the cutoff value for Emax-2 shell was 104.71 kPa, with an AUC (95% CI) of 0.832 (0.777–0.886), sensitivity of 76.5%, specificity of 87.1%, PPV of 87.1%, NPV of 76.5%, and accuracy of 81.5%. Applying the Emax-2 shell cutoff value to 4a, 4b, and 4c subgroups, the results revealed that the optimal diagnostic subgroup was 4a, with an AUC (95% CI) of 0.816 (0.720–0.912). This approach could reduce biopsy procedures by 82 patients (65.6%), alleviating both psychological and financial burdens. Detailed diagnostic metrics for the Emax-2 shell cutoff value across subgroups were presented in Table **5**.

### 3.4 ML Model Interpretation and Hyperparameter Optimization

Hyperparameter optimization for ML algorithms (excluding LR) was performed using a 10-fold cross-validation grid search method.

In this study, the DT model’s maximum tree depth was set to 20; for the RF model, the maximum depth of the tree was not restricted, and the minimum impurity reduction was set to 0.0; for the GNB model, no specific prior probability was set, and the variable smoothing parameter was set to 1e-07; for the SVM model, the regularization parameter C was set to 1.0 with the tolerance of 0.001; for the AdaBoost model, the learning rate was set to 0.1; for the XGBoost model, the L2 regularization coefficient was set to 0.5, the learning rate to 0.3, and the maximum tree depth to 4; for the LightGBM model, the learning rate was set to 2.0 and the maximum tree depth to 20.

These parameter settings collectively enabled the models to fully capture complex data structures, enhanced computational efficiency and prediction accuracy, improved generalization capabilities, and prevented overfitting.

### 3.5 Model Construction and Performance Evaluation

**Fig. (3):**
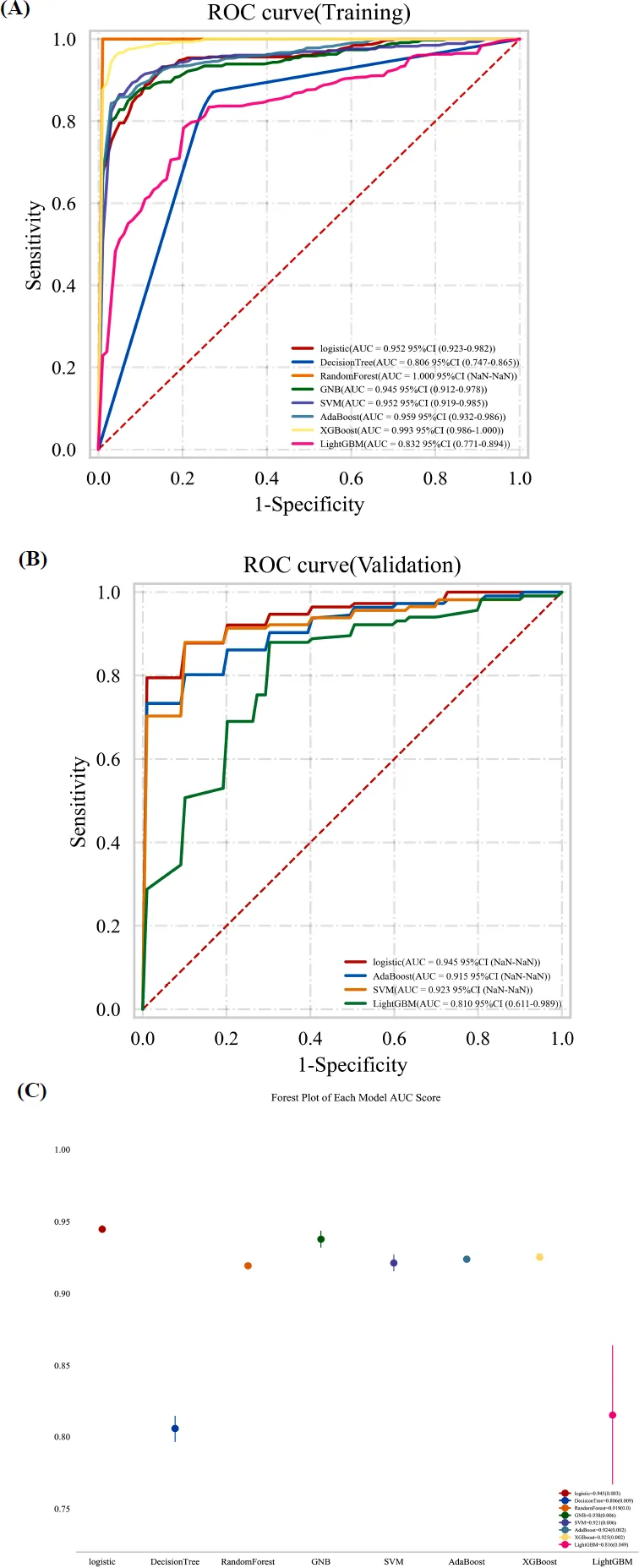
The performance and comparison of eight different prediction models. (**A**) The training set ROC curve; (**B**) The validation set ROC curve; (**C**) Forest plot of AUC values for each model.

Among the eight ML models, the RF algorithm achieved the highest AUC value on the training set but exhibited a significant decline in AUC on the validation set (Fig. **3**), indicating overfitting. The AUC (95% CI) of the RF model on the validation set was 0.921 (0.839–0.999), which showed no statistically significant difference compared to the LR model's AUC (95% CI): 0.947 (0.884–1.000) (P = 0.386). Given the superior stability of the LR model, the LR regression model was selected as the predictive model. The differences in AUC values among the models were not statistically significant. The specific performance metrics of the 8 models were shown in Table **6**. The comparison of ROC curves between the training and validation sets is shown in Fig. (**3A**, **B**). The AUC values of the 8 ML models on the forest plot are shown in Fig. (**3C**).

The LR model was ultimately determined to be the best model after all indicators were thoroughly examined and the possibility of overfitting was taken into account.

### 3.6 Model Interpretability

**Fig. (4):**
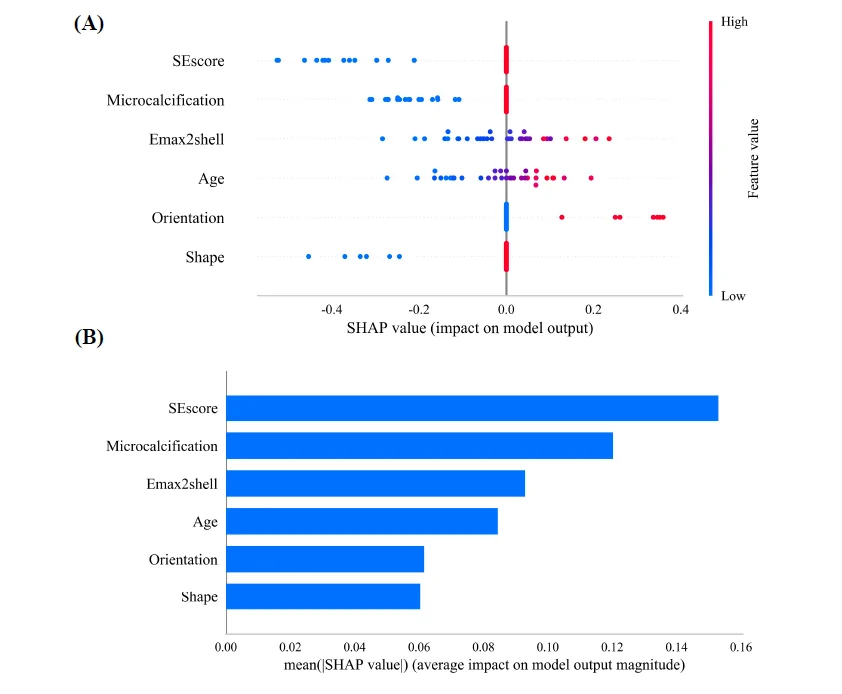
Interpretability analysis of the LR model. (**A**) SHAP honeycomb plot for the LR model's predicted features. (**B**) Feature importance ranking plot for the LR model.

A SHAP honeycomb plot was used in this work for visual analysis to further interpret the LR model's results. As shown in Fig. (**4A**), a patient was represented by each point on the plot. Each feature's impact on the prediction outcome was depicted along the X-axis, while the Y-axis displayed the importance of this feature in the model's decision-making process. Features located higher along the Y-axis had a more significant impact on the final output as they contributed more to the model's prediction. SHAP values were very transparent in terms of how the model came to its decision by giving each feature its own contribution value. It revealed more information about how various predictor variables influenced the model's forecast for each patient. The specific contribution values of the six predictive factors to the model were clearly displayed in the feature importance ranking diagram (Fig. **4B**). Among these, SE score, microcalcification, and Emax-2 shell ranked as the top three most critical predictive features. Finally, force diagrams showed the SHAP values for each sample along with the likelihood of BC.

### 3.7 Nomogram Model

**Fig. (5):**
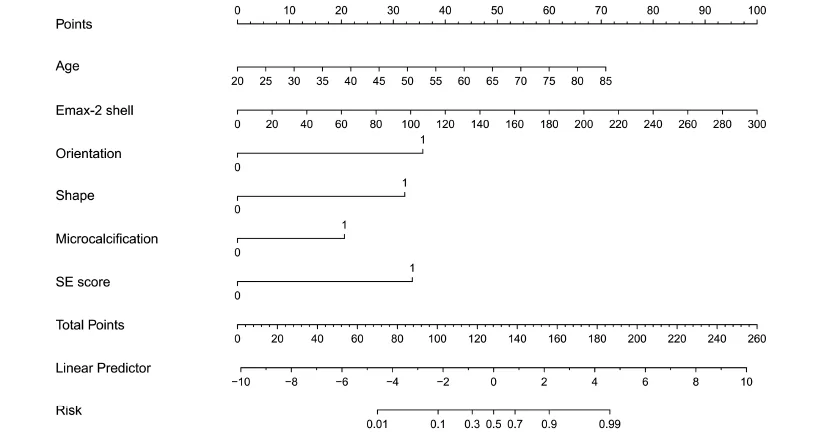
The nomogram Model for Predicting Breast Cancer Risk in BI-RADS 4 Patients. Orientation (0: Parallel growth; 1: Vertical growth); Shape (0: Regular; 1: Irregular); Microcalcification (0: Absent; 1: Present); Strain elasticity Score (0: 1-3 points; 1: 4-5 points).

**Fig. (6):**
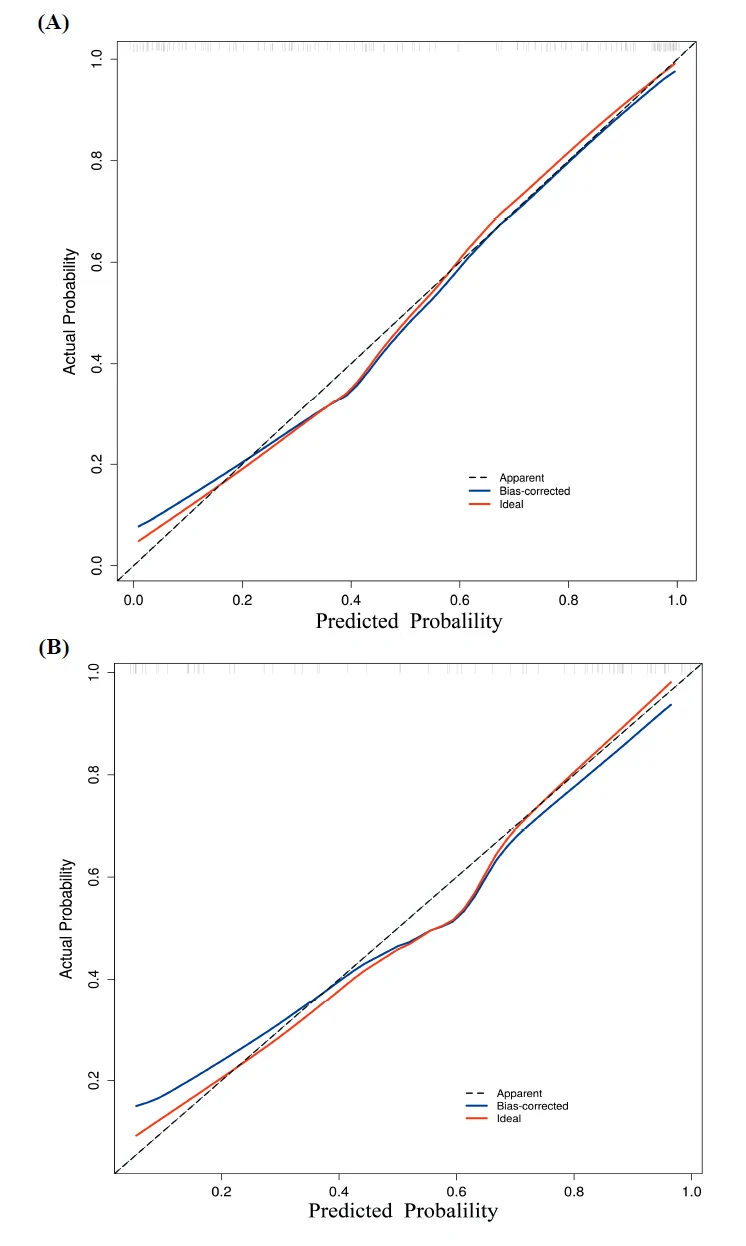
Calibration curves. X-axis: Predicted probability, Y-axis: Actual probability. The red curve represents the ideal prediction curve, while the blue curve indicates the actual probability that occurred. (**A**) The training set calibration curve; (**B**) The validation set calibration curve.

**Fig. (7):**
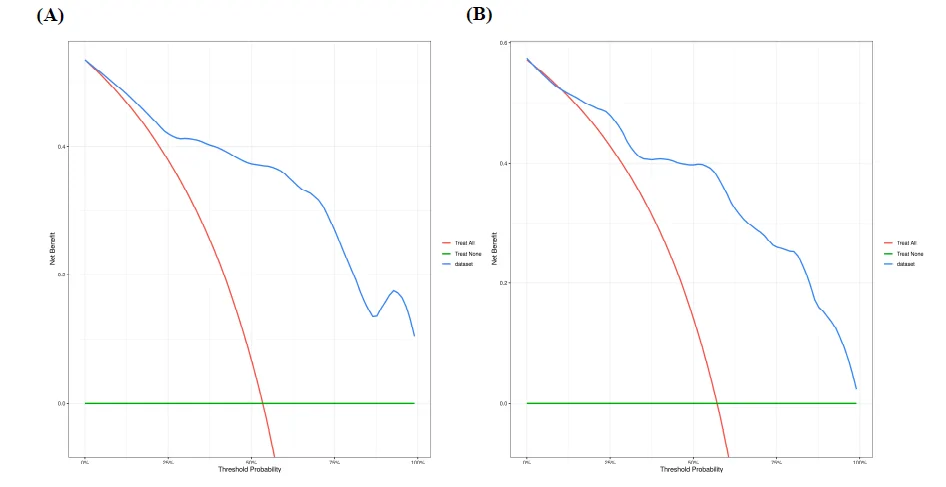
DCA curves. X-axis: Represents the decision threshold, reflecting the minimum risk probability that clinical decision-makers are willing to accept. Y-axis: Measures the net benefit of the model at different thresholds. (**A**) The training set DCA curve; (**B**) The validation set DCA curve.

**Fig. (8):**
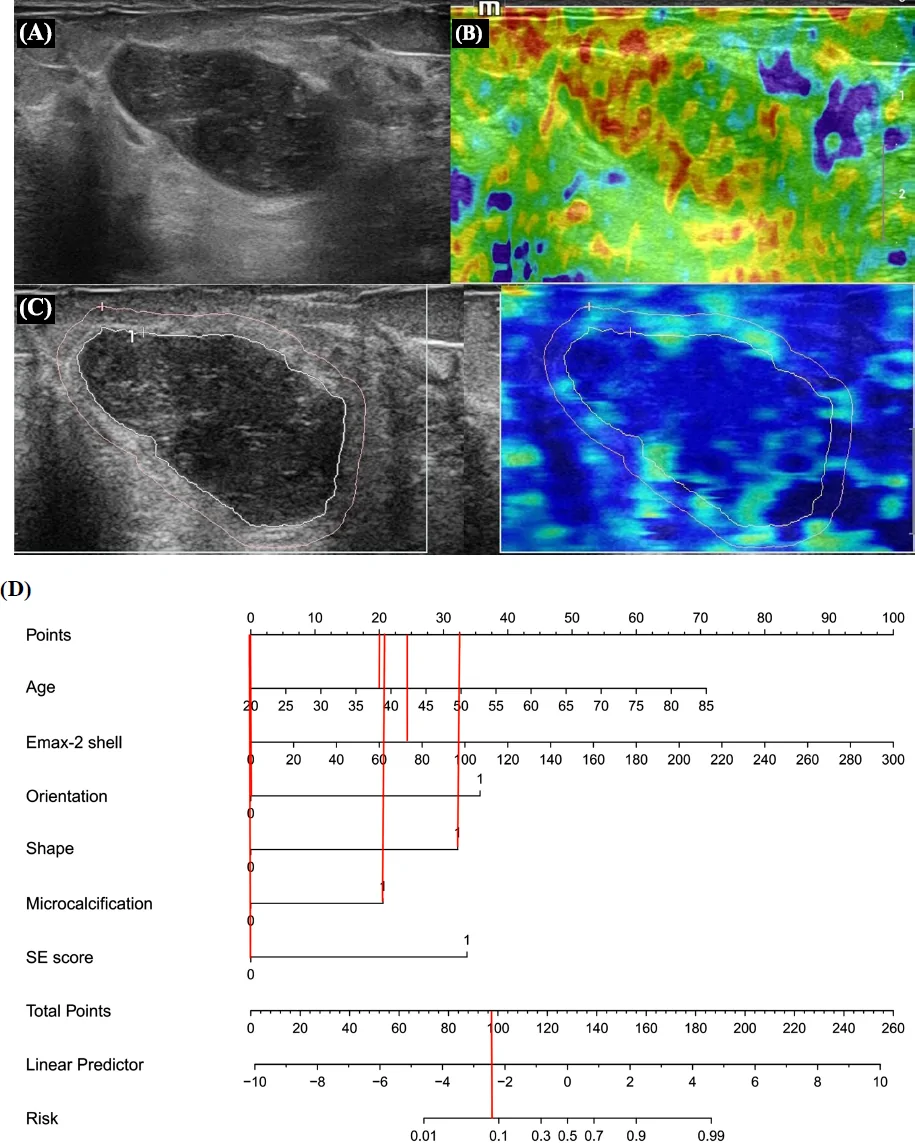
A 38-year-old female patient diagnosed with left breast fibroadenoma. (**A**): 2D ultrasound shows a hypoechoic lesion in the left breast, approximately 29×24×14 mm in size, with clear margin, irregular shape, parallel growth, microcalcification, and posterior acoustic enhancement; (**B**): Strain elastography score: 3; (**C**): Shear wave elastography showed Emax-2 shell: 73.49 kPa, no “stiff rim sign”; (**D**): Total score calculated from Fig. **5**: 20+24+0+21+0+32.5 = 97.5 points, corresponding to a malignancy probability <10%.

**Fig. (9):**
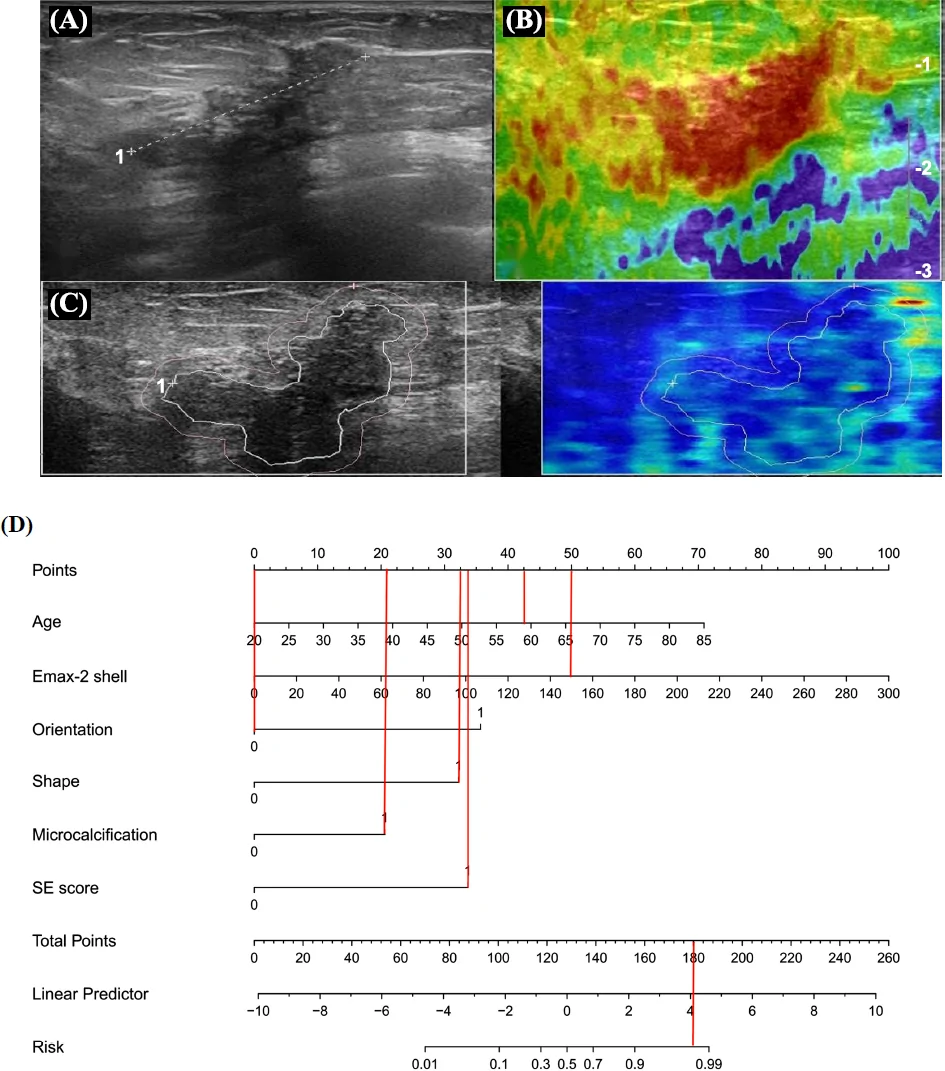
A 59-year-old female patient diagnosed with left breast high-grade ductal carcinoma in situ. (**A**): 2D ultrasound shows a hypoechoic lesion in the left breast, approximately 26×18×10 mm in size, with blurred margin, irregular shape, parallel growth, microcalcification, and posterior acoustic attenuation; (**B**): Strain elastography score of 4 (**C**): Shear wave elastography showed Emax-2 shell: 150.36 kPa, no “stiff rim sign”; (**D**): Total score calculated from Fig. **5**: 42.5 + 50 + 0 + 32.5 + 21 + 34 = 180 points, corresponding to >90% probability of malignancy.

Based on the results of multivariate LR (Table **4**), a nomogram model was constructed (Fig. **5**). Model evaluation: The Hosmer-Lemeshow test indicated good model fit (p-values > 0.05 for both training and validation sets). This demonstrated the model's robust diagnostic performance and stability in distinguishing benign from malignant BI-RADS 4 lesions. Calibration: The calibration curve demonstrated that the predicted curve closely aligned with the ideal curve, indicating high consistency between predicted probabilities and actual probabilities, and high predictive accuracy (Fig. **6**). Clinical Utility: The DCA curve showed that within the threshold range, the model's clinical net benefit significantly exceeded the baseline level, achieving maximum net benefit (Fig. **7**). Figures **8** and **9** illustrated the application of the decision curve analysis to two clinical cases.

## 4 DISCUSSION

This study identified six independent risk predictors associated with BC occurrence in BI-RADS 4 lesion patients through clinical characteristics and conventional ultrasound features combined with elastography features (age, orientation, shape, microcalcification, Emax-2 shell, and SE score). Applying the Emax-2 shell cutoff value to the BI-RADS 4a, 4b, and 4c subgroups revealed optimal diagnostic performance for the 4a subgroup, reducing unnecessary biopsies among patients with BI-RADS 4 lesions. Subsequently, eight ML-based models were developed and compared, selecting the LR model as the optimal risk prediction model and constructing a nomogram. Furthermore, the SHAP algorithm visually demonstrated the contribution of each predictor to the LR model, enhancing its interpretability. The nomogram accurately distinguished benign from malignant BI-RADS 4 lesions, demonstrating clinical utility. These findings validate the predictive capability of the LR model, alleviating patients' psychological and financial burdens while guiding clinicians in formulating personalized treatment plans at an early stage.

The 5-point elasticity scoring system, as well as the strain ratio (SR), are the main quantitative and qualitative indicators of SE. Based on the proportion of different colors, lesions are categorized into five grades; benign lesions have a score of ≤3, while malignant lesions have a score of 4 or 5. Ranjkesh *et al*. [19] also showed that the SE score performed well among all SE-parameters in terms of differentiating benign from malignant breast lesions. The AUC for diagnosing breast lesions rose from 0.95 to 0.97 when the SE score and conventional ultrasound were combined. This coincided with our findings: higher elasticity scores indicated a larger likelihood of malignancy, while lower scores suggested a higher probability of benignity.

In this study, the independent factors that had an effect on the patients with BI-RADS 4 lesions having the occurrence of BC were the Emax-2 shell. When Park *et al*. [20] linearly put a 2mm area of attention inside and outside the tumor’s stroma, the malignant tumors displayed a “quick increase followed by a decrease” pattern. In line with our findings, the highest hardness value was seen in glandular tissue 2-4 mm from the tumor margin. The surrounding tissue of malignant breast tumors typically exhibits higher hardness values than the lesion interior. This phenomenon can be explained by the presence of abnormal collagen in the peritumoral region, combined with cancer cell infiltration into the stroma, which causes a connective tissue proliferation response that makes the peritumoral tissue more rigid [21].

Subtypes of BI-RADS 4 lesions 4a, 4b, and 4c correspond to very low (2-10%), low (10-50%), and high (50-95%) suspicion of malignancy [22]. Therefore, this means that the range of malignant risk probability of BI-RADS 4 lesions corresponds to the BC’s variability of being high. The retrospectively analyzed 216 BI-RADS 4 lesions in our study were found to be 53.2% malignant and 46.8% benign. It would increase the number of needless procedures to perform biopsies on every lesion. Therefore, this study applied the cutoff value of 104.71 kPa for the Emax-2 shell parameter to the BI-RADS 4a, 4b, and 4c groups. The findings indicated that the Emax-2 shell parameter demonstrated the highest diagnostic accuracy and trueness for the BI-RADS 4a group. This suggests that the Emax-2 shell can be utilized in clinical practice to reduce unnecessary biopsies of BI-RADS 4a lesions.

The differences between the Emin and Emin-2 values in this research were not statistically significant (p>0.05). One explanation is that the tumor's interior becomes softer than the surrounding tissue due to necrotic and liquefied areas within malignant lesions, which lower Emin levels. Another possibility is that shear wave energy is attenuated by the tougher surrounding tissue of malignant tumors. As a result, the tumor's shear wave amplitude is reduced, which lowers the Emin value. [23, 24].

The development and progression of tumors are associated with multiple pathological alterations. Increased stromal stiffness around BC tumors results from the combined effects of increased tumor cell numbers, inflammatory cell infiltration, extensive angiogenesis, and connective tissue proliferation.

The extracellular matrix, primarily composed of collagen fibers, not only maintains the matrix's structure, serves as a major component of cells, and sustains cellular homeostasis, but also plays a pivotal role in determining tumor stiffness.

However, the“stiff rim sign” lacks specificity. On one hand, some early-stage BC, such as ductal carcinoma in situ (DICS), exhibited low infiltration and sparse collagen fiber density, presenting with soft or moderate texture [25], where the “stiff rim sign” was not obvious on SWE. Additionally, certain BC subtypes, such as medullary carcinoma, exhibited diffuse tumor cell growth with minimal collagen fiber components, resulting in an indistinct “stiff rim sign” on SWE. On the other hand, certain benign lesions, such as inflammatory nodules, fibromas with calcifications [26], may also exhibit a “stiff rim sign” due to inflammation-mediated fibrous proliferation or fibrin deposition triggered by immune responses. This led to overlapping elastic parameters between benign and malignant lesions, increasing the incidence of false positives and false negatives. This partially explained why the “stiff rim sign” observed in this study was not identified as a potential predictive factor for BC development in patients with BI-RADS 4 lesions.

Age has received a lot of attention as a major risk factor for BC [27]. According to Mazouni *et al*. [28], the incidence of BC rose with age. Consistent with this, our findings indicated that age was a key factor influencing BC development.

Elverici *et al*. [29] identified irregular shape, indistinct borders, and vertical growth pattern as the most likely ultrasound features predicting malignant lesions. The morphological abnormality and vertical growth pattern found in this investigation were consistent with their findings. However, the multivariate regression analysis revealed no statistically significant variations in other ultrasound features, including echo pattern, posterior acoustic characteristics, and blood flow distribution. As a result, these parameters cannot be used as possible indicators of malignancy. Microcalcifications are defined as calcified deposits within human tissue, typically small in diameter (generally considered less than 2 mm, with most ranging between 0.1 and 0.5 mm). They hold significant importance for the early diagnosis of breast cancer. Naseem *et al*. [30] found that microcalcification was closely correlated with invasive breast cancer and HER2 overexpression. In this study, it was indicated that microcalcification constituted an independent predictor of BC development, and this is supported by the corresponding theoretical evidence.

A form of artificial intelligence known as ML is believed to be a dependable and impartial method that has proven to be a beneficial auxiliary in predicting the course of a disease [31]. In this study, a BC risk prediction model based on eight ML algorithms was developed and validated. A comprehensive comparison of various metrics revealed that while the RF model demonstrated outstanding performance on the training set, its metrics declined significantly on the validation set, indicating a risk of overfitting. The LR model exhibited excellent predictive capability on both the training and validation sets, demonstrating strong classification and generalization abilities. However, the risk of overfitting cannot be overlooked. Future research should continue to optimize model parameters, expand sample sizes, and conduct further validation.

In medical practice, the clinical interpretability of ML models is extremely valuable. The SHAP algorithm has the advantage of offering consistent additive feature attributes based on game theory, which makes it possible to explain model predictions both locally and globally, improving the model's transparency and intuitiveness. SHAP analysis in this study intuitively reflected the specific contribution magnitudes of six important predictors to the LR model. This method gives clinicians a new perspective on how the model makes decisions, which improves their level of trust in predictive models.

Combined with SHAP interpretability analysis, the LR-based risk prediction model constructed by this study is highly significant for the clinical treatment of BI-RADS 4 patients. The model, on one hand, can help to characterize high-risk patients by screening out independent risk factors associated with BC occurrence, which can facilitate the development of individualized intervention strategies for timely detection and treatment. On the other hand, the LR model and SHAP interpretability analysis serve as a technique with a transparent, efficient, and visualized tool for clinical practice. In this way, it can help clinicians tackle challenging problems.

Furthermore, the nomogram model constructed based on multivariate LR demonstrated outstanding performance in differential diagnosis, predictive accuracy, and clinical utility. By scoring and visualizing risk, the nomogram serves as an objective diagnostic tool. It can reduce unnecessary biopsies for BI-RADS 4 patients, alleviating psychological and financial burdens while helping clinicians rapidly identify potential cases and optimize clinical decision-making. However, conclusions may be biased by differences in study populations, breast lesion pathology types, sample sizes, and parameter selection. Further refinement is needed.

## 5 LIMITATIONS

Nevertheless, this study possesses several limitations. Initially, as a retrospective analysis, it may be prone to selection bias. Additionally, being a single-center research endeavor with a small cohort size, the results might not be broadly applicable. It is recommended that future research projects integrate a prospective study with a multicenter dataset, in conjunction with sophisticated ML techniques. Such a strategy would contribute to the formulation of more tailored, accurate treatment regimens for patients at an earlier stage.

## CONCLUSION

The BC risk prediction model developed in this study using LR demonstrates excellent diagnostic performance, showing potential as a reliable tool for BC diagnosis. It reduces unnecessary biopsies for BI-RADS 4 patients and provides a scientific basis for clinicians to formulate personalized treatment plans at an early stage. Additionally, it plays a positive role in promoting the application of ML in the field of clinical oncology.

## Figures and Tables

**Fig. (2):**
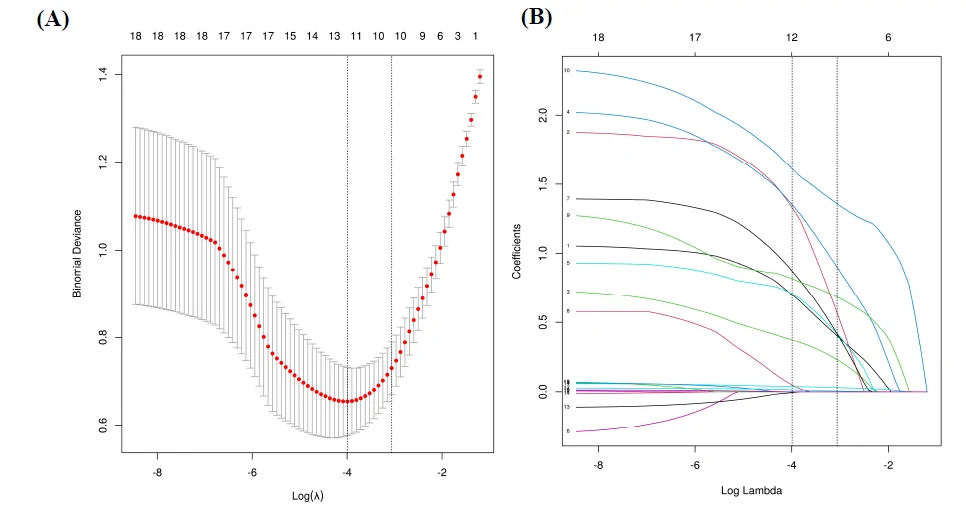
Variable selection process via LASSO regression analysis. (**A**) Cross-validation curve. The point where the dashed line intersects the curve indicates the optimal value selected through 10-fold cross-validation. The model's feature coefficients are shown on the vertical axis. Ten variables in total were chosen with the optimal parameter (lambda. 1se = 0.047). (**B**) Regression coefficient path diagram. The horizontal axis represents log(lambda) values, where lambda is the regularization parameter. Shrinkage becomes more intense as lambda rises, leading to more coefficients shrinking to zero. Each colored curve represents a distinct feature.

**Table 1:** Baseline data comparison of training and validation sets.

**Characteristics**	**Total Date** **(n=216)**	**Training Set** **(n=151)**	**Validation Set** **(n=65)**	**Statistic**	***P***
Age, Mean ± SD	52.00(41.00,61.00)	52.00(38.00,61.00)	51.00(42.00,62.00)	Z=-0.214	0.832
BMI, M (Q1, Q3)	24.60(22.50,26.84)	24.74(22.50,26.89)	24.20(22.30,26.70)	Z=0.387	0.700
Maximum diameter(mm), n (%)	16.00(10.50,22.00)	16.00(11.00,22.00)	15.00(10.00,21.00)	Z=0.847	0.397
Menopausal status, n (%)				X^2^=0.022	0.882
Premenopausal	108(50.00)	75(49.67)	33(50.77)		
Postmenopausal	108(50.00)	76(50.33)	32(49.23)		
Orientation, n (%)				X^2^=0.783	0.376
Parallel	174(80.56)	124(82.12)	50(76.92)		
Non-parallel	42(19.44)	27(17.88)	15(23.08)		
Echo Pattern, n (%)				X^2^=0.506	0.477
Homogeneous	115(53.24)	78(51.66)	37(56.92)		
Heterogeneous	101(46.76)	73(48.34)	28(43.08)		
Shape, n (%)				X^2^=0.446	0.504
Regular	46(21.30)	34(22.52)	12(18.46)		
Irregular	170(78.70)	117(77.48)	53(81.54)		
Margin, n (%)				X^2^=0.847	0.358
Circumscribed	116(53.70)	78(51.66)	38(58.46)		
Non-circumscribed	100(46.30)	73(48.34)	27(41.54)		
Hyperechoic halo, n (%)				X^2^=0.023	0.879
Absent	158(73.15)	110(72.85)	48(73.85)		
Present	58(26.85)	41(27.15)	17(26.15)		
Posterior acoustic, n (%)				X^2^=0.473	0.790
No features	143(66.20)	102(67.55)	41(63.08)		
Shadowing	59(27.32)	40(26.49)	19(29.23)		
Enhancement	14(6.48)	9(5.96)	5(7.69)		
Microcalcification, n (%)				X^2^=0.033	0.857
Absent	101(46.76)	70(46.36)	31(47.69)		
Present	115(53.24)	81(53.64)	34(52.31)		
Stiff Rim Sign, n (%)				X^2^=0.452	0.501
Absent	139(64.35)	95(62.91)	44(67.69)		
Present	77(35.65)	56(37.09)	21(32.31)		
Alder Classification, n (%)				X^2^=2.022	0.155
0-I	181(83.80)	123(81.46)	58(89.23)		
II-III	35(16.20)	28(18.54)	7(10.77)		
Strain Elasticity Score, n(%)				X^2^=0.025	0.876
≤3	88(40.74)	61(40.40)	27(41.54)		
4-5	128(59.26)	90(59.60)	38(58.46)		
Emean, M (Q1, Q3)	34.91(27.04,43.19)	35.17(27.15,43.26)	33.01(26.38,42.08)	Z=0.693	0.489
Emax, M (Q1, Q3)	84.20(65.88,106.65)	84.63(66.03,102.04)	80.49(64.75,116.99)	Z=0.110	0.913
Emin, M (Q1, Q3)	9.32(5.14,15.00)	9.30(4.85,15.28)	9.30(5.760,14.18)	Z=-0.354	0.724
Esd, M (Q1, Q3)	11.19(9.01,14.80)	11.39(8.88,14.39)	11.17(9.80,16.68)	Z=-0.507	0.613
Emean-2 shell, M (Q1, Q3)	36.55(28.87,45.83)	37.20(30.88,45.93)	35.17(25.88,45.63)	Z=1.157	0.248
Emax-2 shell, M (Q1, Q3)	97.71(75.70,127.12)	97.21(77.40,124.64)	97.71(72.170,128.71)	Z=0.261	0.795
Emin-2 shell, M (Q1, Q3)	7.41(3.81,13.15)	7.34(3.39,13.15)	7.41(4.63,13.07)	Z=-0.083	0.935
Esd-2 shell M (Q1, Q3)	15.93(12.03,20.28)	15.93(12.64,20.07)	15.75(11.82,22.30)	Z=0.229	0.820

**Table 2:** Baseline data distribution in the training set.

**Characteristics**	**Total Date** **(n=151)**	**Benign** **(n=71)**	**Maligant** **(n=80)**	**Statictics**	***P***
Age, Mean ± SD	52.00(38.00,61.00)	42.00(34.00,54.00)	58.00(52.00,65.00)	Z=-5.663	<0.001
BMI, M (Q1, Q3)	24.74(22.50,26.89)	24.44(21.80,26.30)	25.00(23.00,27.89)	Z=-1.883	0.060
Maximum diameter(mm), n (%)	16.00(11.00,22.00)	14.00(10.00,20.00)	20.00(13.00,24.40)	Z=-3.219	0.001
Menopausal status, n (%)				X^2^=29.783	<0.001
premenopausal	75(49.67)	52(73.24)	23(28.75)		
postmenopausal	76(50.33)	19(26.76)	57(71.25)		
Orientation, n (%)				X^2^=5.873	0.015
Parallel	124(82.12)	64(90.14)	60(75.00)		
Non-parallel	27(17.88)	7(9.86)	20(25.00)		
Echo Pattern, n (%)				X^2^=1.177	0.278
Homogeneous	78(51.66)	40(56.34)	38(47.50)		
Heterogeneous	73(48.34)	31(43.66)	42(52.50)		
Shape, n (%)				X^2^=29.922	<0.001
Regular	34(22.52)	30(42.25)	4(5.00)		
Irregular	117(77.48)	41(57.75)	76(95.00)		
Margin, n (%)				X^2^=18.900	<0.001
Circumscribed	78(51.66)	50(70.42)	28(35.00)		
Non-circumscribed	73(48.34)	21(29.58)	52(65.00)		
Hyperechoic halo, n (%)				X^2^=17.095	<0.001
Absent	110(72.85)	63(88.73)	47(58.75)		
Present	41(27.15)	8(11.27)	33(41.25)		
Posterior acoustic, n (%)				X^2^=11.026	0.004
No features	102(67.55)	57(80.28)	45(56.25)		
Shadowing	40(26.49)	10(14.09)	30(37.50)		
Enhancement	9(5.96)	4(5.63)	5(6.25)		
Microcalcification, n (%)				X^2^=15.616	<0.001
Absent	70(46.36)	45(63.38)	25(31.25)		
Present	81(53.64)	26(36.62)	55(68.75)		
Stiff Rim Sign, n (%)				X^2^=38.287	<0.001
Absent	95(62.91)	63(88.73)	32(40.00)		
Present	56(37.09)	8(11.27)	48(60.00)		
Alder Classification, n (%)				X^2^=18.188	<0.001
0-I	123(81.46)	68(95.78)	55(68.75)		
II-III	28(18.54)	3(4.22)	25(31.25)		
Strain Elasticity Score, n(%)				X^2^=54.994	<0.001
≤3	61(40.40)	51(71.83)	10(12.50)		
4-5	90(59.60)	20(28.17)	70(87.50)		
Emean, M (Q1, Q3)	36.20±12.06	34.03±12.04	38.13±11.75	t=-2.101	0.037
Emax, M (Q1, Q3)	84.63(66.03,102.04)	73.96(63.10,91.73)	93.38(72.86,121.98)	Z=-3.542	<0.001
Emin, M (Q1, Q3)	9.32(4.80,15.28)	10.09(4.86,15.95)	9.25(4.84,14.70)	Z=0.380	0.705
Esd,M (Q1, Q3)	11.39(8.88,14.39)	10.50(7.91,12.75)	12.29(9.43,18.55)	Z=-2.878	0.004
Emean-2 shell, M (Q1, Q3)	38.79±13.62	33.58±10.44	43.42±14.42	t=-4.806	<0.001
Emax-2 shell, M (Q1, Q3)	97.21(77.40,124.64)	82.74(64.70,98.01)	118.03(86.77,157.53)	Z=-5.872	<0.001
Emin-2 shell, M (Q1, Q3)	7.34(3.39,13.15)	7.43(4.77,12.61)	7.18(2.68,15.08)	Z=0.054	0.958
Esd-2 shell M (Q1, Q3)	15.93(12.64,20.07)	14.05(10.59,16.97)	18.31(14.25,24.85)	Z=-4.625	<0.001

**Table 3:** Univariate logistic regression analysis of breast cancer risk in BI-RADS 4 lesions in the training set.

**Variables**	**β**	**SE**	**Wald**	**OR**	**95%CI**	***P***
Age	0.079	0.015	26.881	1.082	[1.050,1.115]	<0.001
BMI	0.078	0.043	3.291	1.081	[0.994,1.175]	0.070
Maximum diameter	0.065	0.022	8.565	1.068	[1.022,1.115]	0.003
Emean	0.029	0.014	4.228	1.029	[1.001,1.058]	0.040
Emax	0.015	0.005	8.598	1.015	[1.005,1.025]	0.003
Emin	-0.005	0.020	0.060	0.995	[0.956,1.036]	0.807
Esd	0.102	0.034	9.129	1.107	[1.037,1.183]	0.003
Emean-2 shell	0.065	0.016	16.863	1.067	[1.034,1.100]	<0.001
Emax-2 shell	0.028	0.006	21.672	1.028	[1.016,1.040]	<0.001
Emin-2 shell	0.008	0.023	0.122	1.008	[0.964,1.054]	0.726
Esd-2 shell	0.121	0.030	16.152	1.129	[1.064,1.198]	<0.001
Menopausal status	1.914	0.365	27.579	6.783	[3.320,13.858]	<0.001
Orientation	1.114	0.474	5.515	3.048	[1.202,7.724]	0.019
Echo Pattern	0.355	0.328	1.173	1.426	[0.750,2.711]	0.279
Shape	2.632	0.566	21.590	13.902	[4.581,42.195]	<0.001
Margin	1.487	0.350	18.030	4.422	[2.226,8.782]	<0.001
Hyperechoic halo	1.710	0.439	15.195	5.529	[2.340,13.064]	<0.001
Posterior acoustic						
Shadowing	1.335	0.416	10.296	3.800	[1.681,8.589]	0.001
Enhancement	0.460	0.700	0.431	1.583	[0.402,6.241]	0.511
Microcalcification	1.337	0.345	15.039	3.808	[1.937,7.484]	<0.001
Stiff Rim Sign	2.469	0.439	31.596	11.812	[4.994,27.941]	<0.001
Alder Classification	2.323	0.637	13.392	10.303	[2.954,35.932]	<0.001
SE Score	2.882	0.429	45.167	17.850	[7.702,41.367]	<0.001

**Table 4:** Multivariate stepwise regression analysis of breast cancer risk in BI-RADS 4 lesions in the training set.

**Variables**	**β**	**SE**	**Wald**	**OR**	**95%CI**	***P***
Age	0.086	0.023	13.483	1.090	(1.044 - 1.141)	<0.001
Orientation	2.820	0.930	9.203	16.780	(2.713 - 103.799)	0.002
Shape	2.545	0.870	8.561	12.740	(2.317 - 70.061)	0.003
Microcalcification	1.626	0.597	7.425	5.086	(1.579 - 16.384)	0.006
Emax-2 shell	0.026	0.009	8.756	1.027	(1.009 - 1.045)	0.003
SE score	2.660	0.631	17.783	14.298	(4.153 - 49.226)	<0.001

**Table 5:** Comparison of diagnostic performance of Emax-2 Shell (kPa) cutoff value for BI-RADS 4a, 4b, and 4c subgroups.

**Data Set**	**AUC(95%CI)**	**Sensitivity(%)**	**Specificity(%)**	**PPV(%)**	**NPV(%)**	**Accuracy(%)**
BI-RADS 4a	0.816(0.720-0.912)	75.0%	88.2%	68.6%	91.1%	84.8%
BI-RADS 4b	0.774(0.663-0.885)	71.4%	83.3%	97.2%	26.3%	72.7%
BI-RADS 4c	0.676(0.571-0.781)	85.3%	50.0%	96.7%	16.7%	83.3%

**Table 6:** Confusion matrix results of eight machine learning models.

**Data Set**	**Model**	**AUC(95%CI)**	**Accuracy(%)**	**Sensitivity(%)**	**Specificity(%)**	**PPV(%)**	**NPV(%)**	**F1 Score**
Train	LR	0.952(0.923-0.982)	90.2	92.2	87.9	87.9	90.9	0.909
-	DT	0.806(0.747-0.865)	81.0	87.0	74.3	79.4	83.4	0.830
-	RF	1.000(nan-nan)	100.0	100.0	100.0	100.0	100.0	1.000
-	GNB	0.945(0.912-0.978)	89.8	86.5	93.6	94.0	86.0	0.900
-	SVM	0.952(0.919-0.985)	91.4	88.5	94.8	95.1	88.0	0.916
-	AdaBoost	0.959(0.932-0.986)	90.9	85.9	96.5	96.7	85.8	0.909
-	XGBoost	0.993(0.986-1.000)	96.4	96.1	96.8	97.2	95.6	0.966
-	LightGBM	0.832(0.771-0.894)	82.5	79.6	85.9	87.3	79.4	0.828
Valid	LR	0.947(0.884-1.000)	87.1	87.8	86.1	87.7	86.6	0.877
-	DT	0.806(0.687-0.924)	81.0	87.0	74.2	79.5	84.2	0.829
-	RF	0.921(0.839-0.999)	84.7	80.9	89.1	89.8	80.6	0.849
-	GNB	0.941(0.870-0.996)	86.1	82.6	90.1	90.6	82.1	0.863
-	SVM	0.923(0.837-0.998)	85.2	80.9	90.1	90.9	80.7	0.854
-	AdaBoost	0.926(0.850-0.997)	84.3	80.0	89.0	89.8	80.5	0.841
-	XGBoost	0.927(0.847-1.000)	87.5	87.8	87.1	88.8	86.8	0.882
-	LightGBM	0.816(0.692-0.940)	79.1	75.7	83.1	84.4	74.7	0.796

## Data Availability

The data of current study are available from corresponding author, [B.L], on a reasonable request.

## LIST OF ABBREVIATIONS

AdaBoost: Adaptive boosting
AUC: Area under the curve
BC: Breast cancer
BI-RADS: Breast Imaging Reporting and Data System
DT: Decision tree
DCA: Decision curve analysis
DCIS: Ductal carcinoma in situ
E_max_: Maximum Young's modulus hardness value
E_mean_: Mean Young's modulus hardness value
E_min_: Minimum Young's modulus hardness value
E_sd_: Standard deviation of Young's modulus hardness value
Emax-2 shell: The 2 mm shell's Maximum Young's modulus hardness value
Emean-2 shell: The 2 mm shell's Mean Young's modulus hardness value
Emin-2 shell: The 2 mm shell's Minimum Young's modulus hardness value
Esd-2 shell: The Standard deviation of the 2 mm shell's Young's modulus hardness value
GNB: Gaussian naive bayes
HER-2: Human epidermal growth factor receptor
LR: Logistic regression
LASSO: Least absolute shrinkage and selection operator
LightGBM: Light gradient boosting machine
ML: Machine learning
M-STB: Motion stability
MRI: Magnetic resonance imaging
NPV: Negative predictive value
PPV: Positive predictive value
RF: Random forest
ROI: Region of interest
SVM: Support vector machine
SHAP: Shapley additive Explanation
SE: Strain elastography
SWE: Shear wave elastography
UE: Ultrasonic elastography
XGBoost: Extreme gradient boosting

## STANDARDS OF REPORTING

STROBE guidelines were followed.
